# Concentration of heavy metals in street dust: an implication of using different geochemical background data in estimating the level of heavy metal pollution

**DOI:** 10.1007/s10653-020-00726-9

**Published:** 2020-10-10

**Authors:** Sylwia Dytłow, Beata Górka-Kostrubiec

**Affiliations:** Institute of Geophysics, Polish Academy of Sciences, Ks. Janusza 64, 01-452 Warsaw, Poland

**Keywords:** Street dust, Heavy metals, Air pollution, Geochemical background

## Abstract

**Abstract:**

Geochemical background data are used to distinguish between the sources of heavy metal (natural or anthropogenic) and to categorize the level of heavy metal pollution. In this study, we present the results of using different geochemical backgrounds (BG1–BG3) to establish the level of heavy metal pollution in street dust in Warsaw, Poland. We applied individual and collective indicators calculated with respect to the following backgrounds: (1) upper continental crust (UCC) (BG1), (2) the regional geochemical background established for Quaternary surface deposits of the Mazovian region (Poland) (parent geological material occurring in the studied area, Warsaw, Poland) (BG2), and (3) the minimal values of the concentration of heavy metals determined for the real street dust sample collectives from Warsaw (BG3). The assessment of the heavy metals pollution of street dust significantly depended on the background values used in the calculation of individual and collective indicators. Street dust was classified as unpolluted for almost all the heavy metals based on the values of indicators calculated for UCC data. The effect of traffic-related pollution was detected more precisely based on the values of indicators calculated for BG2 and BG3. The naturally elevated concentrations of heavy metals in UCC data can be responsible for the underestimation of pollution impact in street dust. When relatively low concentration of heavy metals is only observed, the application of BG2 or BG3 background data, which better correspond to the geogenic material in street dust, allows to realistically reflect the level of pollution from moving vehicles.

**Graphic abstract:**

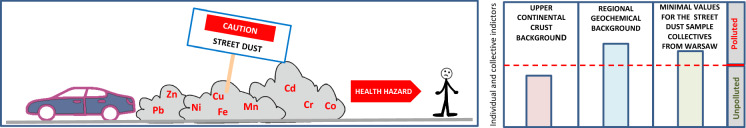

**Electronic supplementary material:**

The online version of this article (10.1007/s10653-020-00726-9) contains supplementary material, which is available to authorized users.

## Introduction

Street dust is one of the most important carriers of heavy metal contaminants usually considered as a valuable indicator of the air quality in an urban environment (Cheng et al. 2018; Huang et al. 2016; Men et al. 2018; Pan et al. 2017; Soltani et al. 2015; Trujillo-González et al. 2016). Non-degradable and toxic heavy metals may cause adverse effects on human health, including cardiovascular, respiratory, and neurodegenerative diseases (Ali et al. 2017; Bi et al. 2018; Chen et al. 2015a; Khademi et al. 2019; Okorie et al. 2012; Zhang et al. 2014). Street dust contains primarily products of vehicle emission and the products of mechanical wear of parts of cars (e.g. tyres, brake discs, and pads) as well as the particles re-suspended from the pavement and unpaved shoulder. In addition to traffic-related particles, the street dust incorporates geogenic material, contaminated soil, and airborne particles settling on the road surface. Therefore, it can be said that street dust is very diverse and has a complex chemical composition in terms of heavy metal (Shi et al. 2011; Li et al. 2013).

Previous studies (Cheng et al. 2018; Men et al. 2018; Soltani et al. 2015; Zgłobicki et al. 2018) have presented an extensive database of absolute concentrations of heavy metals in urban and industrial dust. However, using absolute concentrations to assess the level of pollution requires using the geochemical background, which depends on geological conditions. The application of different geochemical indicators normalized to the background values is a more reliable approach to compare the environmental quality. (Chen et al. 2019; Gope et al. 2018). In general, depending on the method of calculation, the following indicators provide useful information about the level of contamination: (1) geoaccumulation index (*I*_geo_) (Chen et al. 2015b; Dung et al. 2013; Guan et al. 2014; Ghazaryan et al. 2015; Inengite et al. 2015; Karim et al. 2015; Liu et al. 2016; Müller 1969; Ololade 2014; Omotoso and Ojo 2015; Sayadi et al. 2015; Tang et al. 2015; Wang et al. 2015) and contamination factor (CF) (Håkanson 1980; Inengite et al. 2015; Loska et al. 2004) as the level of pollution caused by the individual heavy metal, (2) pollution load index (PLI) as the scale of total heavy metal pollution (Tomlinson et al. 1980), (3) enrichment factor (EF) to detect the source of heavy metal contamination (natural or anthropogenic) (Inengite et al. 2015; Karim et al. 2015; Mohamed et al. 2014), and (4) potential ecological risk index (PERI) as the source of harmful effects on the environment (Pejman et al. 2015; Sayadi et al. 2015; Tang et al. 2015; Wang et al. 2015).

The aforementioned indicators are calculated with respect to baseline values of heavy metals occurring naturally in sediments, soils, and water, and they depend on the composition and mineralogy of local geogenic material (Barbieri 2016; Gałuszka and Migaszewski 2011). One of the commonly used geochemical backgrounds is the data of chemical composition of the UCC, which is formed by igneous, sedimentary, and metamorphic rocks (Gaschnig et al. 2016 and references therein). UCC is composed of highly diverse lithology representing almost every rock type occurring on Earth (Rudnick and Gao 2003; Shaw 1967). Therefore, using UCC can only provide general information about the scale of an anthropogenic impact on the environment. It is also important to consider the regional mineralogical composition of the parent geological material, i.e. information about the baseline natural concentrations of heavy metals which can vary in a large range depending on the geological surface structure occurring in the studied area. For example, the Quaternary surface deposits of the Mazovian region (Poland) are represented mainly by different of sands (loamy and clayey sands) in Czarnowska 1996 and are a primarily parent geogenic component of street dust in Warsaw (Poland).

Geochemical background data are important to distinguish between the natural and anthropogenic sources of heavy metals and to correctly indicate the level of pollution. Many authors (e.g. Barbieri 2015; Gałuszka and Migaszewski 2011; McLennan 2001; Romano et al. 2015) have discussed the difficulty of precisely establishing the geochemical background of chemical elements originating from possible two sources: natural (geogenic material being the product of the natural processes occurring in regional geological structure) and anthropogenic (material generated by human activities). This kind of differences is characteristic for street dust, whose composition originates from both the natural and the anthropogenic sources and undergoes the spatial and temporal (daily and seasonal) variations. In addition, the minimum concentration of heavy metals determined for the real sample collected in the studied area could be alternatively considered as a geochemical background. This background could be used to calculate the indicators assessing the environmental status and the potential ecological risk (Matschullat et al. 2000).

The aim of this study was to take a closer look at approaches to assess the level of heavy metal pollution in the street dust. The primary objective was to test and compare the level of heavy metal pollution established by the individual and collective indicators, such as *I*_geo_, EF, CF, PLI, Er, and PERI, normalized with respect to 3 sets of heavy metal backgrounds: the average heavy metal concentration in UCC (BG1), the regional geochemical background established for Quaternary surface deposits of the Mazovian region (Poland) (parent geological material occurring in the studied area) (BG2), and the minimum values of the concentration of heavy metals determined for the real street dust sample collectives from Warsaw (BG3). We also discussed the implications of using these three backgrounds for the estimation of the degree of environmental pollution and ecological risk caused by traffic-related heavy metals in the urbanized area.

## Materials and methods

### Sampling material, location, and methods

**Fig. 1:**
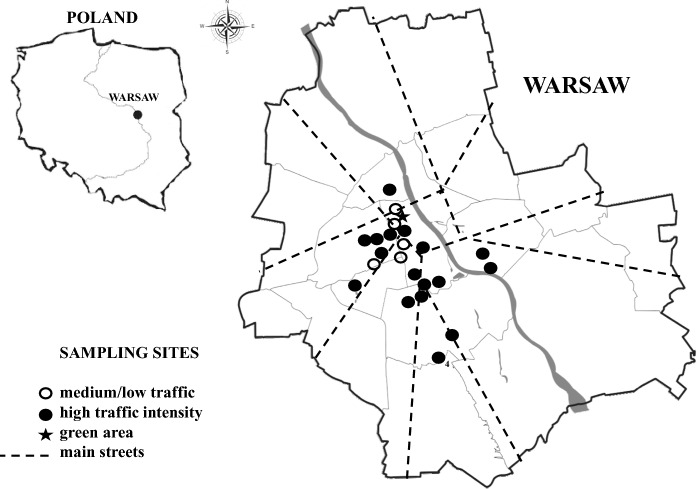
Locations of sampling sites of street dust in Warsaw, Poland

The analysis was conducted on a subset of 23 samples obtained from a set of 248 samples of street dust collected during the summer of 2013 and 2014 from the road surfaces of the city of Warsaw, Poland. The properties of all 248 samples have been evaluated in previous our works in terms of concentration of anthropogenic magnetic particles, their mineralogy, and the state of the domain using magnetic methods (Dytłow and Kostrubiec 2018; Dytłow et al. 2019b). Detailed information about the sampling area and the collection method can be found in Dytłow and Kostrubiec (2018) and Dytłow et al. (2019b). The subset of 23 samples was chosen based on the values of magnetic susceptibility, which is the magnetic parameter approximating the heavy metal load based on the estimation of concentration of the anthropogenic magnetic particles (Bourliva et al. 2018). The sampling sites covered those areas of the city which has different traffic intensities (heavy and low) and different urban activities, such as the central and suburban residential districts with centralized and individual heating systems, as well as the green areas at the very centre and in the outskirts of the city of Warsaw (Fig. 1). It is worth to add that the exhaust and non-exhaust traffic emissions are the dominant sources of pollution particles incorporated to street dust in central districts of Warsaw. Most of the vehicles drive into the city centre and the central parts of the main districts, due to the absence of a “limited traffic zone”. The Warsaw area is less affected by industrial pollution and the heat and power plants emission due to each of the plant has protection systems against the emission of pollutants into the atmosphere. The contribution of the traffic-related sources dominates in the central districts and in the vicinity of the main streets. In the peripheries, there is a strong impact of the area sources of the local heating installations; the central districts have municipal heating installations. The Warsaw agglomeration is dominated by westerly winds.

The samples were naturally air-dried at room temperature and then sieved through a 1-mm mesh to remove waste materials and small stones. Pre-prepared samples (0.15–0.20 g) were digested with ~ 5 mL of 65% HNO_3_ with increasing temperature from 12.5 C min^−1^ to 270 C, holding at 270 C for 15 min in microwave digestion system (Milestone UltraWAVE unit); the samples were cooled back to room temperature. After filtration, the extract was analysed by inductively coupled plasma–mass spectrometry (ICP-MS; ELAN 6100 DRC, Perkin Elmer, USA). The concentrations of the main traffic-related heavy metals (Mn, Fe, Cd, Pb, Zn, Co, Cr, Ni, and Cu) were determined in a standardized chemical laboratory.

### Heavy metal pollution and ecological risk assessment

The following three indicators, commonly used to assess heavy metals, were tested.

The geoaccumulation index is expressed as:

1 $$I_{{{\text{geo}}}} = \log_{2} \left( {\frac{{C_{n} }}{{1.5\; \cdot \;{\text{BG}}_{n} }}} \right)$$

where $C_{n}$ is the concentrate of *n*th chemical element in the sample, ${\text{BG}}_{n}$ is the geochemical background value, and factor 1.5 is the background matrix correlate factor due to natural fluctuation in the content of a given chemical element in the environment with minimum anthropogenic influence (Lu et al. 2009; Müller 1999; Shi et al. 2010; Wei et al. 2009).

The contamination factor described by the following equation:

2 $${\text{CF}}_{n} = \frac{{C_{n} }}{{C_{{{\text{BG}}}} }}$$

is defined as the ratio of the concentrate of *n-*th chemical element in a sample to the individual heavy metal background value *C*_BG_*.*

The enrichment factor is expressed by the following equation:

3 $${\text{EF}} = \frac{{\left( {\frac{{C_{n} }}{{{\rm{RE}}}}} \right)}}{{\left( {\frac{{B_{n} }}{{{\rm{RE}}}}} \right)}}$$

where ${\text{RE}}$ is the concentration of the reference element particularly stable in the soil which does not undergo anthropogenic alteration. Other authors applied Al, Fe, Mn, or Rb (Allen and Rae 1987; Barbieri et al. 2015; Loring 1990; Loring et al. 1995; Balls et al. 1997) as the reference element. In this study, we calculated the EFs using Mn, as it is a conservative element and a major constituent of clay minerals.

The pollution load index is one of the complex indicators, which help in the assessment of the total degree of heavy metal contamination. It is calculated as the geometric mean of individual values of *CFn* (Tomlinson et al. 1980) and is expressed by the equation:

4 $${\text{PLI}} = \sqrt[n]{{{\text{CF}}_{1} \;{\text{CF}}_{2} \ldots {\text{CF}}_{n} }}$$

The potential ecological risk index was tested to assess the potential ecological risk for the environment. It is determined as the sum of the potential ecological risk factors $\left( {E_{r} } \right)$ for the given *n*th element according to the following equation:

5 $${\text{PERI}} = \sum_{n} T_{r}^{n} \;E_{r}^{n}$$

PERI is the multiplication product of toxic-response factor $T_{r}^{n}$ and the contamination factor *CFn* of the *n*th element. $T_{r}^{n}$ is 30, 5, 5, 5, 2, 1, and 1 for Cd, Cu, Pb, Ni, Cr, Mn, and Zn, respectively (Håkanson 1980).

The numerical data of *I*_geo_, ER, Er, and PERI are indicative of different levels of pollution and ecological risk; Table 1S presents their classified values (Supplementary material).

The information regarding the background values of an individual heavy metal or other reference data such (e.g. the concentration of reference elements) is necessary for the calculation of individual indicators and potential ecological risk factors. We tested all the indicators for three backgrounds (values of BG1–BG3 are presented in Table 2S); the UCC background after Taylor and McLennan (1985) (BG1), regional geochemical background for Quaternary fluvioglacial sands (BG2), and concentration corresponding to the street dust sample exhibiting the lowest concentration of heavy metals (BG3). The regional geochemical background is the concentration of heavy metals established for Quaternary deposits in the Mazovian region occurred at the depth of 80–120 cm and represented by fluvioglacial slightly loamy sand (Czarnowska 1996). BG3 is established as the minimum concentration of heavy metals for the in-field collected sample selected from the set of 248 dust samples obtained from road surfaces in Warsaw. We state that BG3 reflects properties of material naturally occurring on the roadside surface, which contributes to the street dust as the very local geogenic material.

### Statistical analysis

Descriptive statistic was applied in order to graphically depict the groups of numerical data through their quartiles. The top and bottom ranges of the box represent the 25th and 75th percentiles, respectively, the bend inside the box is the median value, and the square mark is the mean value. The whiskers indicate variability outside the upper (95%) and lower (5%) quartiles. The additional values (minimum and maximum values) are marked as the cross points. All the graphs were statistically analysed and plotted by employing software Origin 8.6. OriginLab (OriginLab Corp. USA).

## Results

### Concentration of heavy metals

**Fig. 2:**
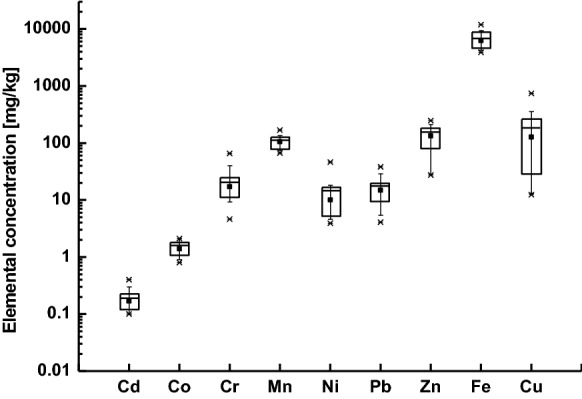
The box-and-whisker plot of concentration of traffic-related heavy metals in street dust from Warsaw, Poland

Figure 2 shows the variations in the concentration of individual traffic-related heavy metals in the street dust. The median values revealed significant diversity in the concentration of Fe, Zn, Cu, and Mn predominating among the studied heavy metals and followed by Pb, Cr, Ni, Co, and Cd; (Cd(0.183) < Co(1.781) < Ni(16.99) < Pb(17.06) < Cr(20.27) < Mn(110.1) < Zn(149.9) < Cu(184.3) < Fe(6801), see Table 3S). Moreover, the setting of elements in the increasing order of the coefficient of variation Mn(0.21) < Co(0.25) < Fe(0.31) < Pb(0.53) < Cd(0.59) < Cr(0.77) < Ni(0.87) < Cu(1.29) indicates variability in the concentration between individual sites from low and moderate to high.

According to Polish legal regulation, only the content of Cu in 5 street dust samples was higher than the limits of this metal in the soil for residential land uses (Regulation of Ministry of Environment 2016). However, compared to much more restrictive regulation standards than in Poland, e.g. the Dutch regulation (Ministerie van Volkshuisvesting 2000), in addition to Cu content, two metals Zn and Ni had the concentrations higher than standard values. The concentration of Cu exceeded the upper standard limit in 12 locations, and Zn and Ni in 10 and 1 locations, respectively. Due to the high degree of toxicity, Cd, Cr, and Pb rank among the most toxic metals, harmful for human health. The concentrations of Cd, Cr, and Pb in Warsaw’s street dust samples were significantly lower than the standard upper limit. Therefore, the Cd, Cr, and Pb posed a relatively health risk to the city residents. A significant hazard to human health may come from Cu and Zn, which high concentrations were found in almost half of the studied locations in the city.

### Assessment of level of heavy metal pollution

#### Geoaccumulation index

**Fig. 3:**
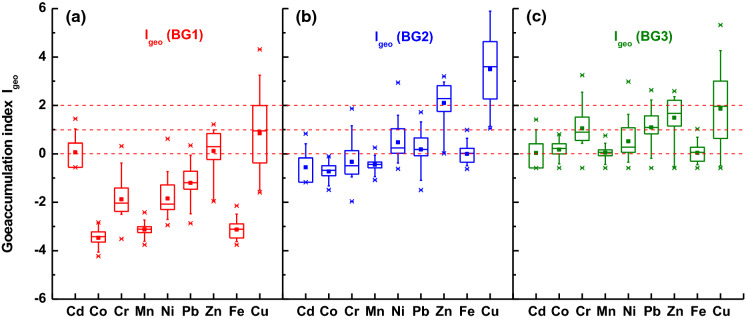
Assessment of level of pollution by *I*_geo_ calculated for the following background values: **a** upper continental crust (UCC) (BG1), **b** regional geogenic material (BG2), and **c** for the minimal concentration of heavy metals in the collection of street dust from Warsaw, Poland (BG3)

Most of the values for *I*_geo_ calculated with respect to BG1 (Fig. 3a) were below 0, which corresponds to an unpolluted level (Table 1S) (Müller, 1979). However, the median values of Cd, Zn, and Cu obtained in the following order: 0 ≤ *I*_geo_ ≤ 1 indicated unpolluted to moderately polluted level. The *I*_geo_(BG2) showed unpolluted to moderately polluted level for Ni and Pb (Fig. 3b). The highest level of pollution with *I*_geo_ (BG2) median value of approximately 4 and 2 was obtained for Cu and Zn, respectively. The median values of *I*_geo_(BG3) of > 0 and < 2 indicated a moderate level of pollution for all the elements (Fig. 3c). It is worth emphasizing that, regardless of background data, the highest pollution level was found for 2 elements: Zn and Cu. We also obtained an interesting result for Ni, in which case the value of *I*_geo_ (BG1) was about −2, where the value of *I*_geo_ (BG2) was slightly above 0. This is the case when the UCC (BG1) is used for assemblage samples with a relatively low concentration of individual elements (small anthropogenic impact). Relatively high values in UCC interfere with the proper assessment of the real anthropogenic impact. On the contrary, when UCC is applied to a sample enriched in a particular element (with concentration many times higher than in UCC), then the anthropogenic impact is easy to observe. From the comparison of values of *I*_geo_ calculated for different backgrounds, it can be observed that only *I*_geo_ (BG2) and *I*_geo_ (BG3) show a significant level of pollution. Conversely, *I*_geo_ (BG1) does not reflect the real anthropogenic impact on the environment due to a significantly higher concentration of the UCC background than that of the values in BG2.

#### Enrichment factor

**Fig. 4:**
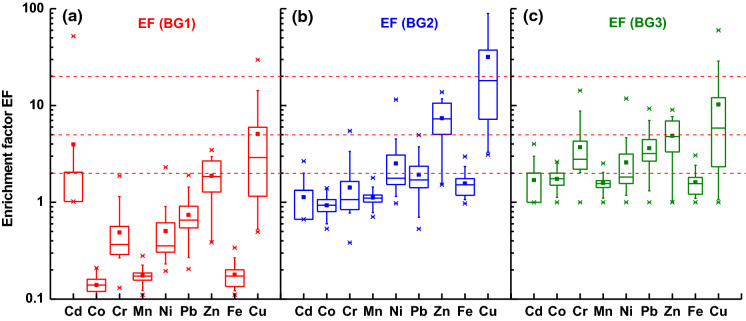
Assessment of level of pollution by enrichment factor (EF) calculated for the following background values: **a** upper continental crust (UCC) (BG1), **b** regional geogenic material (BG2), and **c** for the minimal concentration of heavy metals in the collection of street dust from Warsaw, Poland (BG3)

Enrichment factor is used to distinguish the contribution of heavy metals originating from crustal material and anthropogenic sources. The median values of EF(BG1) for Co, Cd, Cr, Mn, Ni, Pb, Zn, and Fe (Fig. 4a) were < 2 indicating that their origin was from crustal materials or natural processes. Only for Cu, the medians of EF(BG1) exceeded a value of 2 suggesting the influence of anthropogenic activities. The median values of EF(BG2) for Cd, Co, Cr, Mn, Ni, Pb, and Fe were about 1 and < 2 (Fig. 4b). Only Zn and Cu with extremely high median values (7 and 13) demonstrated a significant anthropogenic input. In the case of BG3, the majority of heavy metals exhibit minimal to moderate enrichment in non-crustal material (Fig. 4c); however, EF(BG3) for Cu and Zn exhibited lower values than that of EF(BG2). Consequently, the enrichment of street dust in anthropogenic origin Zn and Cu changed from significant (for BG2) to moderate (for BG3). This could be due to the specific properties of the local material contributing to Warsaw’s street dust, which is enriched in Cu and Zn mainly of traffic origin. Therefore, a relatively high concentration of Zn and Cu (significantly higher than in BG2) was obtained even in city areas, where low traffic intensity occurs. This agrees with the results of other studies conducted on street dust from Poland (Adamiec et al. 2016), which revealed that concentration of tracers of non-exhaust emission, i.e. Cu and Zn, originating from brake and tyre wear were very high especially in fine fractions (< 20 µm) of all types of material sampled in road environment (street dust, soils, and sludge).

#### Pollution load index

**Fig. 5:**
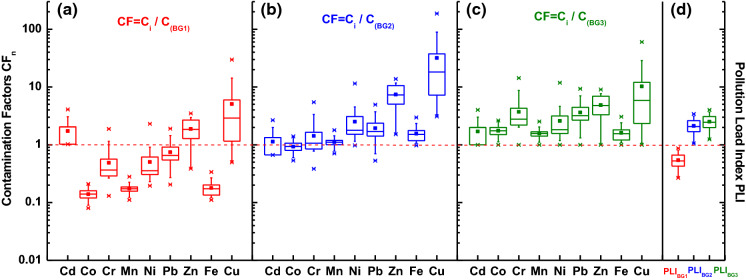
Assessment of level of pollution by contamination factor (CF) and pollution load index (PLI) calculated for the following background values: **a** upper continental crust (UCC) (BG1), **b** regional geogenic material (BG2), and **c** for the minimal concentration of heavy metals in the collection of street dust from Warsaw, Poland (BG3)

A comparison of contamination factors calculated for BG1, BG2, and BG3 revealed significant differences (Fig. 5a–c) in the medians of CF for the individual heavy metals. The median values of CF(BG1) for Co, Cr, Mn, Ni, Pb, and Fe (Fig. 5a) were < 1 indicating low metal pollution level and only median values of CF > 1 for Cd, Zn, and Cu were attributive of anthropogenic origin with a degree of moderately pollution. The distribution of median values of CF(BG2) (Fig. 5b) shows great variability: no metal enrichment for Cd, Co, Cr, and Mn; considerable contamination for Pb, Ni, and Fe; and very high contamination for Zn and Cu. The CF(BG3) (Fig. 5c) seems to be more consistent revealing considerable contamination for most of the metals, the exception is Zn and Cu, which revealed very high contamination. The median values of PLI(BG1) were < 1, which according to Table 1S indicates a lack of heavy metal pollution and natural origin of the heavy metals. More than 2 times higher values of PLI (2.1 and 2.5 for BG2 and BG3, respectively) indicate a moderate load of heavy metal pollution.

#### Potential ecological risk

**Fig. 6:**
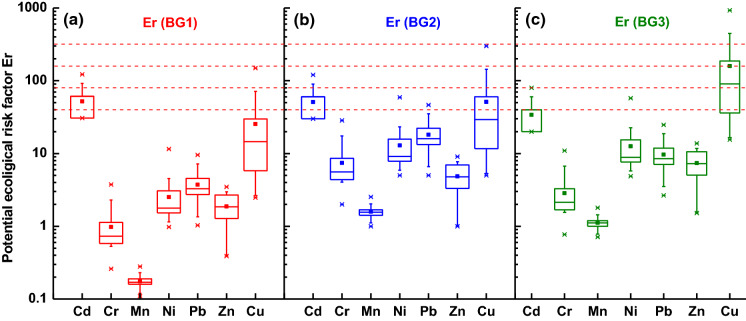
Potential ecological risk factor (Er) of each heavy metal calculated with respect to the following background values: **a** upper continental crust (UCC) (BG1), **b** regional geogenic material (BG2), and **c** for the minimal concentration of heavy metals in the collection of street dust from Warsaw, Poland (BG3)

Potential ecological risk factor (Fig. 6a–c) was calculated for Cd, Cr, Mn, Ni, Pb, Zn, and Cu due to their increased toxicity when compared with other heavy metals. In general, the distribution trend of Er for all backgrounds (Fig. 6) was similar, displaying low ecological risk for most of the heavy metals. An exception to this was Cd and Cu: Cd with a median value for Er(BG1) and Er(BG2) of > 40 showed average risk and Cu with Er(BG3) about 150 revealed considerable risk for the environment.

**Fig. 7:**
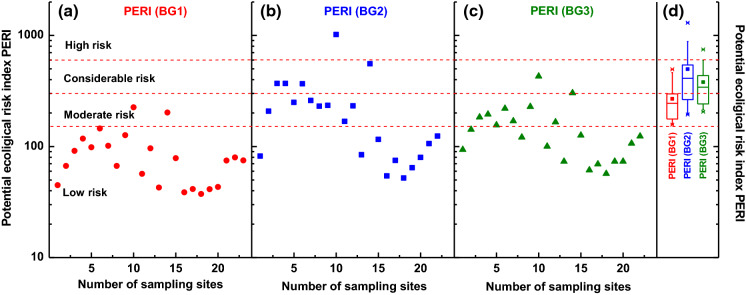
Potential ecological risk index (PERI) for individual sampling sites calculated for the following background values: **a** upper continental crust (UCC) (BG1), **b** regional geogenic material (BG2), and **c** for the minimal concentration of heavy metals in the collection of street dust from Warsaw, Poland (BG3). **d** Total potential ecological risk index for three backgrounds

Figure 7a–c shows the potential ecological risk index calculated separately for each of 23 sampling sites. The analysis of median values of PERI(BG1) indicates (Fig. 7a) a moderate risk for only 2 locations, whereas the low risk was assigned to 21 locations. The results were more diverse for PERI(BG2) (Fig. 7b) distributing samples in following 11:7:4:1 proportions in 4 levels of ecological risk: low, moderate, considerable, and high, respectively. Whereas for PERI(BG3) the sampling sites were distributed in three levels of ecological risk: low, moderate, and considerable in proportions 14:7:2, respectively.

**Fig. 8:**
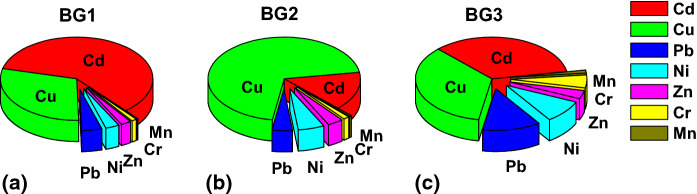
Contribution of potential ecological risk factor (Er) calculated for individual heavy metals to the total value of potential ecological risk index for street dust in Warsaw, Poland

In an environment, traffic-related heavy metals generally occur in the form of complex mixtures. Therefore, the potential adverse risks rather than an individual effect of heavy metal may be of greater interest. Figure 8 shows the percentages of Er for heavy metals referred to the total PERI presented in Fig. 7d. The distribution of Er values for individual metals showed an interesting pattern regardless of the background. At least 90% of PERI was dominated by the contribution of Cd, Cu, Zn, Pb, and Ni, whereas the rest of the heavy metal load was distributed mainly between Mn and Cr. Depending on the background, the contributions of Cd, Cu, Zn, Pb, and Ni to PERIs were different. A similar pattern was observed for BG1 and BG3, for which potential ecological risk coming from Cd was the highest among the 7 heavy metals which accounted for 59–33% of the total PERIs, respectively. Background BG2 revealed the greater contribution of Cu (70%) to the total PERIs compared to BG1 and BG3. The second highest contribution to the total PERIs came from Cu, which accounted for 30–35% for BG1 and BG3, respectively.

## Discussion

In this study, the median values obtained for traffic-related heavy metals in street dust from Warsaw were found to be low when compared with big European cities and other cities from around the world (Cheng et al. 2018; Men et al. 2018; Soltani et al. 2015); also the variability in median values showed differences in order and degree. The concentration of Pb, Fe, Zn, and Cu in Warsaw is at least one order lower than that of most cities from well-developed countries: i.e. Newcastle in the UK, Barcelona in Spain, Kavala in Greece, Toronto in Canada, and Massachusetts in the USA, as well as in comparison to Chinese cities (Table 3S). In contrast, the concentration of Ni in Warsaw’s street dust is relatively low and comparable with those obtained from the streets of Lahore (in Pakistan) and Nanjing (in China). The concentration of Cd, Cr, Ni, Pb, and Zn in Warsaw differ from those obtained for another Polish city (Lublin) (Zgłobicki et al., 2018). For street dust from Lublin was obtained significantly higher values of mean content of Cd, significantly lower for Cu and similar concentrations of Ni. Adamiec (2017) provided the concentration of Cu and Zn in the fine granulometric fraction (grain-size of < 20 µm) of street dust from Warsaw; these values were 3 times higher for Cu and one order of magnitude higher for Zn compared to data obtained in our study. These differences are due to the fact that about 90% of all the heavy metals from non-exhaust emission are contributed to the fraction with a grain size of < 20 µm (Adamiec et al. 2016). This agrees with the results of our previous study (Dytłow et al. 2019b) on the magnetic and chemical properties of different granulometric fractions of street dust in Warsaw. It was found that the two finest fractions with a diameter of “0.1 mm–71 μm” and “  < 71 μm” had the smallest contribution to the total mass of dust, but contained the highest concentrations of Fe, Cr, Cd, Co, Zn, Pb, Ni, Mn, Cu, and Al. In the grain size fraction of < 71 µm, the heavy metal concentrations were at least twice higher than that of coarser granulometric fractions, with peaks observed up to 16 times higher for Cu. These considerations had two important implications in terms of comparison of data between different sampling sites as well as their adverse effect on human health. Due to the significantly different contribution of heavy metals to the individual granulometric fractions of dust, all comparisons should include the information about the grain size of the fraction for which the absolute concentration of heavy metals was determined. Unfortunately, it is a common practice to compare the concentration of heavy metals between different cities without considering granulometric fractions, which could cause misinterpretations in the estimation of pollution level. Epidemiological study (e.g. Harrison and Yin 2000) indicated that in addition to the toxicity of heavy metals, mass, size, number, and surface area of particles transferring hazardous pollutant are the most important determinant of health impact. Ultra-fine particles exert more significant physiological effect (fine particles produce enhanced inflammatory responses) than coarse particle even in the same mass.

Two individual indicators, i.e. *I*_geo_ and EF were primarily used to distinguish the natural and anthropogenic sources of heavy metals present in sediments, rocks, and soils. Further, these indices were adapted to pollution studies of other materials, i.e. street dust. The physico-chemical nature of street dust is lesser known than sediments, rocks, and soils, and street dust properties could change dynamically (i.e. seasonal changes). Hence, their implementation to analyse street dust should be done very carefully. The heavy metal impact on the composition of street dust in Warsaw was found to be very diverse depending on the background data applied to calculate *I*_geo_ and EF. In general, the smallest pollution influences were observed for the normalization of *I*_geo_ and EF by UCC background (BG1); the values of *I*_geo_ for BG2 and BG3 were elevated when compared with that of BG1, resulting in the elevation of the anthropogenic impact. In the cases of *I*_geo_(BG1) and EF(BG1), street dust was less affected by exanimated heavy metals; however, in the case of Zn and Cu, their anthropogenic input was noticeable. The anthropogenic impact observed for Cu and Zn, even when applying UCC (BG1), is due to relatively high concentrations of these elements.

The general observation is that the application of BG2 and BG3 is a better choice in street dust, as the heavy metal input is not masked by high background concentrations. The use of UCC (BG1) values in the anthropogenic impact evaluation can be valid only if the concentration of heavy metals are several times higher than that of the geochemical background. More specifically, naturally elevated levels of heavy metals in UCC can be responsible for the underestimation of the level of traffic-related heavy metal pollution which in consequence does not give a reliable assessment of real anthropogenic input of individual elements to street dust. A similar level of pollution obtained for *I*_geo_(BG2) and *I*_geo_(BG3) could be explained by their comparable background concentrations, which are significantly lower than that in UCC.

PLI requires the use of an individual index such as CF which assessing the level of individual heavy metal. No significant difference in values of PLI(BG2) and PLI(BG3) was observed as both values of PLI classified the street dust of Warsaw as moderately polluted. The underestimated level of pollution assessed by PLI(BG1) is clearly caused by the application of UCC background in order to calculate individual CF values. The application of UCC to PLI normalization provides the real level of pollution in 2 cases: when the geogenic material incorporated in street dust is naturally enriched in heavy metals originated from UCC or/and when traffic-related metals significantly (many times) exceed background concentrations. However, when a low concentration of heavy metals is observed, as in this study, only the application of background concentrations, which better correspond to the geogenic concentration of an individual element in street dust, allows to realistically categorize the level of pollution. An alternative solution may be to use the passive samplers instead of dust collected directly from the surface of the road. Our previous study (Dytłow and Górka-Kostrubiec 2018) was concerned on the evaluation of traffic-related pollution using passive samplers which exhibited good properties to accumulate the traffic-related pollution and well-specified baseline values obtained before exposition on pollution. PLI values calculated for passive samplers with respect to their background, i.e. the concentration of an individual heavy metal determined before exposure to pollution, were 10–200% higher than that calculated in this study for street dust using BG3. This result once again confirms that knowledge of the geogenic contribution of individual heavy metals in street dust is crucial for the assessing of pollution levels using individual and comprehensive indicators.

As a result, the PERI, which collectively evaluates the pollution risk of many heavy metals through a toxic-response Er factor, is well suited for the estimation of street dust. The median values of Er (Fig. 6) show a wide variation depending on the background, but with the exception of Cu, none of the metals poses an ecological hazard. In contrast, the PERI calculated separately for each sampling site (Fig. 7) classifies their potential ecological risk as moderate, considerable to high for almost half the locations. This result indicates that a complex urban environment with very strong local variability in traffic-related emissions requires meticulous analysis made separately for each of the sampling sites to detect ecological hazards. Therefore, it seems to be reasonable to apply a combined approach to calculate the pollution indicators and spatial distribution, e.g. geographic information systems (GIS) in the study of urban environmental air quality by the estimation of pollution level using street dust.

It is well known that the first step in the analysis of pollution is to define the concentration of individual elements which occur naturally without human influence (known as the background level) in a given environment (Adriano 2001; Desaules 2012; Gałuszka and Migaszewski 2011; Matschullat et al. 2000; Reimann and Garrett 2005; Tack et al. 1997). The estimation of the background level is often a complicated task in an urban environment because street dust, by definition, is a material composed of natural and anthropogenic components. Given these difficulties, the good quality background values should represent the lithogenic deposits occurring on the surface, because it is highly possible that this natural material would be incorporated in street dust. Our study showed that due to the complexity and local nature of background values, the responsible approach is a detailed street dust sampling of the study area. It is important to specify sampling sites, which represents locally changing conditions influencing the concentrations and transport of traffic-related pollution, i.e. city centre, downtown areas, rotary areas, different traffic intensities, type of car movement (braking, accelerating), and building density. Because we provide statistically representative wide spectrum set of street dust samples, it is reasonable to choose from this set, the minimum values of concentration of heavy metals as a background for calculation of indicators. Although there are limitations of performing chemical analysis for a few hundred of samples, it is very important to screen the samples (first-approach selection) based on other low-cost and fast-measured parameter. Our research and experience (e.g. Dytłow et al. 2019a) shows that magnetic susceptibility has the potential to be used to screen the great assemblage samples as it is strongly correlated with the concentration of traffic-related heavy metals.

## Conclusion

The comparison of indicators normalized by different backgrounds reveals significant differences in assessing the level of pollution estimated on the basis of street dust. Indicators calculated according to the local background and the minimum concentration of the heavy metal for street dust sample allows detecting even low anthropogenic input of traffic-related heavy metals. However, at the same time, normalization by UCC background shows a deficiency of anthropogenic enrichment. Only for extremely polluted areas, the values of UCC will be a suitable background; however, for less polluted areas, the anthropogenic influence will be masked by high background concentrations.

Generally, this study showed a higher level of pollution based on the values of BG2 ((Cd) 0.098 mg/kg d.m. < (Co) 10 < (Ni) 20 and (Pb) 20 < (Cu) 25 < (Cr) 35 < (Zn) 71 < (Mn) 600 < (Fe) 35,000) and BG3 ((Cd) 0.1 mg/kg d.m. < (Co) 0.8 < (Ni) 3.9 < (Pb) 4.1 < (Cr) 4.6 < (Cu) 12.4 < (Zn) 27.4 < (Mn) 66.5 < (Fe) 3886); thus, we can conclude that these backgrounds detect the effect of traffic-related pollution more precisely. The calculation according to BG2 could bring valuable knowledge as it considers great variability of the site- and region-dependent surface deposits usually incorporated to street dust. Nonetheless, in the case of complicated and complex local lithology, the pollution assessment could be done based on the minimum values obtained for street dust samples. In order to avoid artificial anthropogenic impact coming from local lithological material, BG3 could be responsibly applied if we provide representative assemblage samples, reflecting locally changing conditions influencing the concentration of heavy metals.

As a result of our research, we recommend using the following backgrounds under the following circumstances. BG1 can be used when the concentrations of heavy metal of anthropogenic origin are several times higher than that of the geochemical background. The use of UCC, which is assumed to be the same for the entire earth's crust, makes the results more comparable to other studies. Local geochemical background (BG2) provides the data of chemical elements present in the geogenic material physically incorporated in street dust. Therefore, gives us the information of real anthropogenic contribution to the final concentration of heavy metals in street dust. BG3 precisely reflects the anthropogenic contribution if it is based on sufficient sample collection. Each applied indicator was originally invented for a different research material than street dust and secondary applied to street dust. Therefore, for the assessment of heavy metal pollution in street dust, we recommend using indicators collectively.

## Electronic supplementary material

Below is the link to the electronic supplementary material.

Supplementary file1 (DOCX 32 kb)

## References

[CR1] Adamiec E (2017). Chemical fractionation and mobility of traffic-related elements in road environments. Environmental Geochemistry and Health.

[CR2] Adamiec E, Jarosz-Krzemińska E, Wieszała R (2016). Heavy metals from non-exhaust vehicle emissions in urban and motorway road dusts. Environmental Monitoring and Assessment.

[CR3] Adriano, D.C. (2001). *Trace elements in terrestrial environments: Biogeochemistry, bioavailability and risks of metals* (pp. 65). (2nd edition), Springer-Verlag, New York.

[CR4] Ali MU, Liu G, Yousaf B, Abbas Q, Ullah H, Munir MAM, Fu B (2017). Pollution characteristics and human health risks of potentially (eco) toxic elements (PTEs) in road dust from metropolitan area of Hefei. China Chemosphere.

[CR5] Allen JRL, Rae JE (1987). Late Flandrian shoreline oscillations in the Severn Estuary: A geomorphological and stratigraphical reconnaissance. Philosophical Transactions of Royal Society B Biological Sciences.

[CR6] Balls PW, Hull S, Miller BS, Pirie JM, Proctor W (1997). Trace metal in Scottish estuarine and coastal sediments. Marine Pollution Bulletin.

[CR7] Barbieri M, Nigro A, Sappa G (2015). Soil contamination evaluation by enrichment factor (EF) and geoaccumulation index (Igeo). Senses and Sciences.

[CR8] Bi C, Zhou Y, Chen Z, Jia J, Bao X (2018). Heavy metals and lead isotopes in soil, road dust and leafy vegetables and health risks via vegetable consumption in the industrial areas of Shanghai, China. Science of the Total Environment.

[CR9] Bourliva A, Kantiranis N, Papadopoulou L, Aidona E, Christophoridis C, Kollias P, Evgenakis M, Fytianos K (2018). Seasonal and spatial variations of magnetic susceptibility and potentially toxic elements (PTEs) in road dusts of Thessaloniki city, Greece: A 1 year monitoring period. Science of the Total Environment.

[CR10] Chen H, Teng Y, Lu S, Wang Y, Wang J (2015). Contamination features and health risk of soil heavy metals in China. Science of the Total Environment.

[CR11] Chen RH, Wang BQ, Wang ZB, Yao S (2015). The pollution character analysis and risk assessment for metals in dust and PM_10_ around road from China. Biomedical and Environmental Sciences.

[CR12] Chen X, Guo M, Feng J, Liang S, Han D (2019). Characterization and risk assessment of heavy metals in road dust from a developing city with good air quality and from Shanghai, China. Environmental Science and Pollution Research.

[CR13] Cheng Z, Chen L, Li H, Lin J, Yang Z, Yang Y, Xu X, Xian J, Shao J, Zhu X (2018). Characteristics and health risk assessment of heavy metals exposure via household dust from urban area in Chengdu, China. Science of the Total Environment.

[CR14] Christoforidis A, Stamatis N (2009). Heavy metal contamination in street dust and roadside soil along the major national road in Kavala’s region, Greece. Geoderma.

[CR15] Czarnowska K (1996). Total content of heavy metals in parent rocks as reference background levels of soils. Roczniki Gleboznawcze.

[CR16] Desaules A (2012). Critical evaluation of soil contamination assessment methods for trace metals. Science of the Total Environment.

[CR17] Dung TTT, Cappuyns V, Swennen R, Phung NK (2013). From geochemical background determination to pollution assessment of heavy metals in sediments and soils. Reviews in Environmental Science and Bio/Technology.

[CR18] Duzgoren-Aydin NS (2007). Sources and characteristics of lead pollution in the urban environment of Guangzhou. Science of the Total Environment.

[CR19] Dytłow, S., Górka-Kostrubiec, B. (2018). Comparison of traffic-related pollution level using street dust and passive dust samplers. Conference Proceedings 3rd International Conference on Atmospheric Dust—DUST2018, Published by Digilabs S.a.s. 10.14644/dust.2018.006.

[CR20] Dytłow S, Górka-Kostrubiec B (2019). Effective and universal tool for evaluating heavy metals—passive dust samplers. Environmental Pollution.

[CR21] Dytłow S, Winkler A, Górka-Kostrubiec B, Sagnotti L (2019). Magnetic, geochemical and granulometric properties of street dust from Warsaw (Poland). Journal of Applied Geophysics.

[CR22] Gałuszka A, Migaszewski Z (2011). Geochemical background: An environmental perspective. Mineralogia.

[CR23] Gaschnig RM, Rudnick RL, McDonough WF, Kaufman AJ, Valley JW, Hu Z, Gao S, Beck ML (2016). Compositional evolution of the upper continental crust through time, as constrained by ancient glacial diamictites. Geochimica et Cosmochimica Acta.

[CR24] Ghazaryan KA, Gevorgyan GA, Movsesyan HS, Ghazaryan NP, Grigoryan KV (2015). The evaluation of heavy metal pollution degree in the soils around the zangezur copper and molybdenum combine, Rome, Italy. International Journal of Environmental, Chemical, Ecological, Geological and Geophysical Engineering.

[CR25] Gope M, Masto RE, George J, Balachandran S (2018). Tracing source, distribution and health risk of potentially harmful elements (PHEs) in street dust of Durgapur, India. Ecotoxicology and Environmental Safety.

[CR26] Górka-Kostrubiec B, Jeleńnska M, Król E (2014). Magnetic signature of indoor air pollution: Household dust study. Acta Geophysica.

[CR27] Górka-Kostrubiec B (2015). The magnetic properties of indoor dust fractions as markers of air pollution inside buildings. Building and Environment.

[CR28] Guan Y, Shao Ch, Ju M (2014). Heavy metal contamination assessment and partition for industrial and mining gathering areas. International Journal of Environmental Research and Public Health.

[CR29] Håkanson L (1980). An ecological risk index for aquatic pollution control. A sedimentological approach. Water Research.

[CR30] Harrison RM, Yin J (2000). Particulate matter in the atmosphere: Which particle properties are important for its effects on health?. Science of the Total Environment.

[CR31] Huang J, Li F, Zeng G, Liu W, Huang X, Xiao Z, Wu H, Gu Y, Li X, He X, He Y (2016). Integrating hierarchical bioavailability and population distribution into potential eco-risk assessment of heavy metals in road dust: A case study in Xiandao district, Changsha city, China. Science of the Total Environment.

[CR32] Inengite AK, Abasi CY, Walter C (2015). Application of pollution indicators for the assessment of heavy metal pollution in flood impacted soil. International Research Journal of Pure and Applied Chemistry.

[CR33] Kam W, Liacos JW, Schauer JJ, Delfino RJ, Sioutas C (2012). Size-segregated composition of particulate matter (PM) in major roadways and surface streets. Atmospheric Environment.

[CR34] Karim Z, Qureshi BA, Mumtaz M (2015). Geochemical baseline determination and pollution assessment of heavy metals in urban soils of Karachi, Pakistan. Ecological Indicators.

[CR35] Khademi H, Gabarrón M, Abbaspour A, Martínez-Martínez S, Faz A, Acosta JA (2019). Environmental impact assessment of industrial activities on heavy metals distribution in street dust and soil. Chemosphere.

[CR36] Li Z, Feng X, Li G, Bi X, Zhu J, Qin H, Dai Z, Liu J, Li Q, Sun G (2013). Distributions, sources and pollution status of 17 trace metal/metalloids in the street dust of a heavily industrialized city of central China. Environmental Pollution.

[CR37] Liu R, Wang M, Chen W, Peng C (2016). Spatial pattern of heavy metals accumulation risk in urban soils of Beijing and its influencing factors. Environmental Pollution.

[CR38] Loring DH (1990). Lithium—a new approach for the granulometrical normalization of trace metal data. Marine Chemistry.

[CR39] Loring DH, Naes K, Dahle S, Matishow GG, Illin G (1995). Arsenic, trace metals, and organic micro contaminants in sediments from the Pechora Sea, Russia. Marine Geology.

[CR40] Loska K, Wiechulab D, Korus I (2004). Metal contamination of farming soils affected by industry. Environment International.

[CR41] Matschullat J, Ottenstein R, Reimann C (2000). Geochemical background—Can we calculate it?. Environmental Geology.

[CR42] McLennan SM (2001). Relationships between the trace element composition of sedimentary rocks and upper continental crust. Geochemistry, Geophysics, Geosystems.

[CR43] Men C, Liu R, Xu F, Wang Q, Guo L, Shen Z (2018). Pollution characteristics, risk assessment, and source apportionment of heavy metals in road dust in Beijing, China. Science of the Total Environment.

[CR44] Mohamed TA, Mohamed MAK, Rabeiy R, Ghandour MA (2014). Application of pollution indices for evaluation of heavy metals in soil close to phosphate fertilizer plant, Assiut, Egypt. Assiut University Bulletin for Environmental Researches.

[CR45] Müller G (1969). Index of geoaccumulation in sediments of the Rhine River. GeoJournal.

[CR46] Okorie A, Entwistle J, Dean JR (2012). Estimation of daily intake of potentially toxic elements from urban dust and the role of oral bioaccessibility testing. Chemosphere.

[CR47] Ololade IA (2014). An assessment of heavy-metal contamination in soils within auto-mechanic workshops using enrichment and contamination factors with geoaccumulation indexes. Journal of Environmental Protection.

[CR48] Omotoso OA, Ojo OJ (2015). Assessment of some heavy metals contamination in the soil of river Niger floodplain at Jebba, central Nigeria. Water Utility Journal.

[CR49] Pan H, Lu X, Lei K (2017). A comprehensive analysis of heavy metals in urban road dust of Xi'an, China: Contamination, source apportionment and spatial distribution. Science of the Total Environment.

[CR50] Pant P, Harrison RM (2013). Estimation of the contribution of road traffic emissions to particulate matter concentrations from field measurements: A review. Atmospheric Environment.

[CR51] Pejman A, Gholamrez Nabi B, Saeedi M, Baghvanda A (2015). A new index for assessing heavy metals contamination in sediments: A case study. Ecological Indicators.

[CR52] Reimann C, Garrett RG (2005). Geochemical background–concept and reality. Science of the Total Environment.

[CR53] Romano E, Bergamin L, Croudace IW, Ausili A, Maggi C, Gabellini M (2015). Establishing geochemical background levels of selected trace elements in areas having geochemical anomalies: The case study of the Orbetello lagoon (Tuscany, Italy). Environmental Pollution.

[CR54] Rudnick RL, Gao S (2003). Composition of the continental crust, treatise on geochemistry. Treatise on Geochemistry.

[CR55] Sayadi MH, Shabani M, Ahmadpour N (2015). Pollution index and ecological risk of heavy metals in the surface soils of Amir-Abad area in Birjand City, Iran. Health Scope.

[CR56] Sezgin N, Ozcan HK, Demir G, Nemlioglu S, Bayat C (2003). Determination of heavy metal concentrations in street dusts in Istanbul E-5 highway. Environment International.

[CR57] Shaw DM, Reilly GA, Muysson JR, Pattenden GE, Campbell FE (1967). An estimate of the chemical composition of the Canadian precambrian shield. Canadian Journal of Earth Sciences.

[CR58] Shi G, Chen Z, Bi C, Teng J, Wang L, Xu S (2010). Comprehensive assessment of toxic metals in urban and suburban street deposited sediment (SDSs) in the biggest metropolitan area of China. Environmental Pollution.

[CR59] Shi GT, Chen ZL, Bi CJ, Wang L, Teng JY, Li YS, Xu SY (2011). A comparative study of health risk of potential metals in urban and suburban road dust in the most populated city of China. Atmospheric Environment.

[CR60] Soltani N, Keshavarzi B, Moore F, Tavakol T, Lahijanzadeh AR, Jaafarzadeh N, Kermani M (2015). Ecological and human health hazards of heavy metals and polycyclic aromatic hydrocarbons (PAHs) in road dust of Isfahan metropolis, Iran. Science of the Total Environment.

[CR61] Tack FMG, Verloo MG, Vanmechelen L, Van Ranst E (1997). Baseline concentration levels of trace elements as a function of clay and organic carbon contents in soils in Flanders (Belgium). Science of the Total Environment.

[CR62] Tang Q, Li Y, Xu Y (2015). Land suitability assessment for post-earthquake reconstruction: A case study of Lushan in Sichuan, China. Journal of Geographical Sciences.

[CR63] Tomlinson DL, Wilson JG, Harris CR, Jeffrey DW (1980). Problems in the assessment of heavy-metal levels in estuaries and the formation of a pollution index. Helgoländer Meeresunters.

[CR64] Trujillo-González JM, Torres-Mora MA, Keesstra S, Brevik EC, Jiménez-Ballesta R (2016). Heavy metal accumulation related to population density in road dust samples taken from urban sites under different land uses. Science of the Total Environment.

[CR65] Wang Z, Wang Y, Chen L, Yan C, Yan Y, Chi Q (2015). Assessment of metal contamination in coastal sediments of the Maluan Bay (China) using geochemical indicators and multivariate statistical approaches. Marine Pollution Bulletin.

[CR66] Wei B, Jiang F, Li X, Mu S (2009). Spatial distribution and contamination assessment of heavy metals in urban road dusts from Urumqi, NW China. Microchemical Journal.

[CR67] Zgłobicki W, Telecka M, Skupiński S, Pasierbińska A, Kozieł M (2018). Assessment of heavy metal contamination levels of street dust in the city of Lublin, Poland. Environmental Earth Sciences.

[CR68] Zhang M, Lu X, Chen H, Gao P, Fu Y (2014). Multi-element characterization and source identification of trace metal in road dust from an industrial city in semi-humid area of Northwest China. Journal of Radioanalytical and Nuclear Chemistry.

