# Supplementary material for: Concentration of heavy metals in street dust: an implication of using different geochemical background data in estimating the level of heavy metal pollution
Source: Environ Geochem Health. 2020 Oct 10;43(1):521–35. doi: 10.1007/s10653-020-00726-9 (PMC7847877; doi:10.1007/s10653-020-00726-9)
Supplement: Supplementary file 1 — Supplementary file1 (DOCX 32 kb) [file 10653_2020_726_MOESM1_ESM.docx]

**Supplementary material**

Table 1S.

| Indicator | Range of value | Pollution category |
| --- | --- | --- |
| Enrichment factor (EF) ^[1], [2]^ | EF < 2  2 ≤ EF ≤ 5  5 ≤ EF ≤ 20  5 ≤ EF ≤ 20  EF ≥ 40 | Deficiency to minimal enrichment  Moderate enrichment  Significant enrichment  Very high enrichment  Extremely high enrichment |
| Index of geoaccumulation (I_geo_) ^[3]^ | Igeo < 0  0 ≤ Igeo < 1  1 ≤ Igeo < 2  2 ≤ Igeo < 3  3 ≤ Igeo < 4  4 ≤ Igeo < 5  Igeo ≥ 5 | Uncontaminated/unpolluted (class 0)  Unpolluted/moderately (class 1)  Moderately (class 2)  Moderately/heavily (class 3)  Heavily (class 4)  Heavily/extremely(class 5)  Extremely (class 6) |
| Contamination factor (CF) ^[4]^ | CF < 1  1 ≤ CF ≤ 3 3 ≤ CF ≤ 6  CF > 6 | low metal enrichment  Moderately contamination  Considerable contamination  Very high contamination |
| Pollution Load Index (PLI) ^[4]^ | PLI < 1  1< PLI< 2  2< PLI< 3  PLI >3 | no pollution  moderate  heavy  extremely heavy |
| Ecological risk factor (Er) ^[4]^ | Er < 40  40 ≤ Er < 80  80 ≤ Er < 160  160 ≤ Er < 320  Er ≥ 320 | Low  Moderate  Considerable  High  Very high |
| Potential ecological risk index (RI) ^[4]^ | RI<150  150 ≤ RI < 300  300 ≤ RI < 600  RI ≥ 600 | Low  Moderate  Considerable  High risk |

Table 2S The concentrations [mg/kg] of heavy metals in street dust from Warsaw and other cities.

| **Country** | **Sites** | **Co** | **Cr** | **Cu** | **Fe** | **Mn** | **Ni** | **Pb** | **Zn** | **Hg** | **Cd** | **Reference** |
| --- | --- | --- | --- | --- | --- | --- | --- | --- | --- | --- | --- | --- |
|  |  | **[mg /kg]** | | | | | | | | | |  |
| **Poland** | **Warsaw**  **(median)** | **1.781** | **20.27** | **184.3** | **6801** | **110.1** | **16.99** | **17.06** | **149.9** |  | **0.183** | **This study** |
| Afghanistan | Jalalabad | 4 | 25 | 30 |  | 190 | 31 | 33 | 96 |  | 0.7 | [5] |
| Afghanistan | Kabul | 9 | 38 | 44 |  | 253 | 66 | 29 | 123 |  | 1.2 | [5] |
| Canada | Toronto |  | 198 | 162 | 48,235 | 1407 | 59 | 183 | 233 |  |  | [6] |
| China | Chengdu |  | 83 | 190 |  |  | 53 | 123 | 675 |  |  | [7] |
| China | Beijing |  | 92 | 83 | 29,745 | 554 | 33 | 61 | 281 | 0.2 |  | [8] |
| China | Guangzhou |  | 176 | 192 |  | 540 | 41 | 388 | 1777 | 0.2 |  | [9] |
| Colombia | Villavicencio |  | 26 | 213 |  |  | 22 | 467 | 210 |  |  | [10] |
| Egypt | Delta region |  | 86 | 102 | 32,050 | 503 | 39 | 307 | 1839 |  |  | [11] |
| Greece | Kavala |  | 196 | 124 |  |  | 58 | 301 | 272 | 0.1 | 0.2 | [12] |
| Iran | Shiraz |  | 67 | 136 | 20,255 | 439 | 78 | 116 | 404 | 1.1 | 0.5 | [13] |
| Iran | Isfahan | 14 | 82 | 182 |  |  | 70 | 393 | 707 |  | 2.1 | [14] |
| Iran | Tehran |  | 34 | 225 | 47,936 | 1215 | 35 | 257 | 873 |  | 10.7 | [15] |
| Pakistan | Rawalpindi |  | 93 | 157 |  |  | 48 | 146 | 890.0 |  | 8.4 | [16] |
| Pakistan | Lahore | 3 | 20 | 13 |  | 93 | 8 | 170 | 196 |  | 2.3 | [17] |
| Spain | Barcelona |  |  | 1332 |  |  | 58 | 248 | 1572 |  | 3.0 | [18 |
| Switzerland | Zurich |  |  | 3547 |  |  | 504 | 247 | 2183 |  | 10.0 | [18] |
| Turkey | Tokat |  | 30 | 29 |  | 285 | 65 | 149 | 63 |  | 3.0 | [19] |
| Poland | Lublin |  | 86 | 82 |  |  | 16 | 44 | 241 |  |  | [20] |
| UK | Newcastle |  |  | 132 |  |  | 26 | 992 | 421 |  |  | [21] |
| USA | Massachusetts |  | 95 | 105 |  |  |  | 73 | 240 |  |  | [22] |

**Table references**

1. Duzgoren-Aydin, N.S., 2007. Sources and characteristics of lead pollution in the urban environment of Guangzhou. Sci Total Environ. 385, 182–195, doi: 10.1016/j.scitotenv.2007.06.047.
2. Sezgin N., Ozcan H.K., Demir G., Nemlioglu S., Bayat C. 2003. Determination of heavy metal concentrations in street dusts in Istanbul E-5 highway. Environ Int. 29, 979–985, doi: 10.1016/S0160-4120(03)00075-8.
3. Müller G. 1969. Index of geoaccumulation in sediments of the Rhine River. GeoJournal. 2, 108–118.
4. Håkanson, L., 1980. An ecological risk index for aquatic pollution control. A sedimentological approach Water Res. 14, 975-1001.
5. Jadoon W.A., Khpalwak W., Chidya R.C.G., Abdel-Dayem S.M.M.A., Takeda K., Makhdoom M.A., Sakugawa H. 2018. Evaluation of levels, sources and health hazards of road-dust associated toxic metals in Jalalabad and Kabul cities, Afghanistan. Arch Environ Contam Toxicol 74, 32–45.
6. Nazzal Y., Rosen M.A., Al-Rawabdeh A.M. 2013. Assessment of metal pollution in urban road dusts from selected highways of the greater Toronto area in Canada. Environ Monit Assess 185, 1847–1858.
7. Cheng, Z., Chen, L., Li, H., Lin, J., Yang, Z., Yang, Y., Xu, X., Xian, J., Shao, J., Zhu, X., 2018. Characteristics and health risk assessment of heavy metals exposure via household dust from urban area in Chengdu, China. Sci. Total Environ. 619-620, 621–629.
8. Men, C., Liu, R., Xu, F., Wang, Q., Guo, L., Shen, Z., 2018. Pollution characteristics, risk assessment, and source apportionment of heavy metals in road dust in Beijing, China. Sci. Total Environ., 612, 138-147.
9. Huang M., Wang W., Chan C.Y., Cheung K.C., Man Y.B., Wang X., Wong M.H. 2014. Contamination and risk assessment (based on bioaccessibility via ingestion and inhalation) of metal (loid) s in outdoor and indoor particles from urban centers of Guangzhou, China. Sci Total Environ. 479, 117–124.
10. Trujillo-González J.M., Torres-Mora M.A., Keesstra S., Brevik E.C., Jiménez-Ballesta R. 2016. Heavy metal accumulation related to population density in road dust samples taken from urban sites under different land uses. Sci. Total Environ. 553, 636–642.
11. Khairy M.A., Barakat A.O., Mostafa A.R., Wade T.L. 2011. Multielement determination by flame atomic absorption of road dust samples in delta region, Egypt. Microchem. J. 97, 234–242.
12. Christoforidis, A., Stamatis, N. 2009. Heavy metal contamination in street dust and roadside soil along the major national road in Kavala’s region, Greece. Geoderma 151, 257–263.
13. Li N., Kang Y., Pan W., Zeng L., Zhang Q., Luo J. 2015. Concentration and transportation of heavy metals in vegetables and risk assessment of human exposure to bioaccessible heavy metals in soil near a waste-incinerator site, South China. Sci. Total Environ. 521, 144–151.
14. Soltani, N., Keshavarzi, B., Moore, F., Tavakol, T., Lahijanzadeh, A.R., Jaafarzadeh, N., Kermani, M., 2015. Ecological and human health hazards of heavy metals and polycyclic aromatic hydrocarbons (PAHs) in road dust of Isfahan metropolis, Iran. Sci. Total Environ. 505, 712–723.
15. Xue M., Yang Y., Ruan J., Xu Z. 2012. Assessment of noise and heavy metals (Cr, Cu, Cd, Pb) in the ambience of the production line for recycling waste printed circuit boards. Environ. Sci. Technol. 46, 494–499.
16. Abbasi M.N., Tufail M., Chaudhry M.M. 2013. Assessment of heavy elements in suspended dust along the Murree highway near Capital City of Pakistan. World Appl. Sci. J. 12, 1266–1275.
17. Mohmand J., Eqani S.A.M.A., Fasola M., Alamdar A., Mustafa I., Ali N., Liu L., Peng S., Shen H. 2015. Human exposure to toxic metals via contaminated dust: bio-accumulation trends and their potential risk estimation. Chemosphere 132,142–151.
18. Amato F., Pandolfi M., Moreno T., Furger M., Pey J., Alastuey A., Bukowiecki N., Prevot A.S.H., Baltensperger U., Querol X. 2011. Sources and variability of inhalable road dust particles in three European cities. Atmos. Environ. 45, 6777–6787.
19. Kurt-Karakus P.B. 2012. Determination of heavy metals in indoor dust from Istanbul, Turkey: estimation of the health risk. Environ. Int. 50, 47–55.
20. Zgłobicki W., Telecka M., Skupiński S., Pasierbińska A., Kozieł M. 2018. Assessment of heavy metal contamination levels of street dust in the city of Lublin, Poland. Environ. Earth Sci. 77, 1–11.
21. Pourkhabbaz A., Pourkhabbaz H. 2012. Investigation of toxic metals in the tobacco of different Iranian cigarette brands and related health issues. Iran J. Basic Med. Sci. 15, 636–644.
22. Apeagyei E., Bank, M.S., Spengler J.D. 2011. Distribution of heavy metals in road dust along an urban-rural gradient in Massachusetts. Atmos. Environ. 45, 2310–2323.
